# Checklist of the Empidoidea of Finland (Insecta, Diptera)

**DOI:** 10.3897/zookeys.441.7154

**Published:** 2014-09-19

**Authors:** Jere Kahanpää

**Affiliations:** 1 Finnish Museum of Natural History, Zoology Unit, P.O. Box 17, FI-00014 University of Helsinki, Finland

**Keywords:** Checklist, Finland, Diptera, Atelestidae, Brachystomatidae, Dolichopodidae, Hybotidae, Empididae

## Abstract

An updated checklist of the Atelestidae, Brachystomatidae, Dolichopodidae, Empididae and Hybotidae (Diptera) recorded from Finland is presented. The genera with uncertain placement within superfamily Empidoidea (= the *Iteaphila* group) are also included in this paper.

## Introduction

Empidoidea (dance flies and long-legged flies) is one of the most diverse lineages of Diptera with some 13,000 species currently recognized worldwide (Pape et al. 2011) and many more still undescribed. The superfamily is generally accepted as monophyletic, but its subdivision into families, subfamilies and tribes is still being debated.

The families Atelestidae, Brachystomatidae, Hybotidae and Empididae were traditionally all included in Empididae *sensu lato*. Chvála (1983) split the empidids into five families: Empididae *sensu stricto*, Hybotidae, Atelestidae, Dolichopodidae and Microphoridae. Sinclair and Cumming (2006) analyzed the whole superfamily Empidoidea, elevated Brachystomatidae to a full family and transferred Microphorinae to the long-legged flies (Dolichopodidae). Two of the Finnish genera, *Anthepiscopus* Becker and *Iteaphila* Zetterstedt, remain unassigned on family level (Sinclair and Cumming 2006, Shamshev and Sinclair 2009). The classification proposed by Sinclair and Cumming (2006) is adopted for this checklist.

The world fauna of the Empidoidea has recently been catalogued by Yang et al. (2006, 2007). The Finnish species were last listed by Kahanpää and Winqvist (2005) for Atelestidae, Brachystomatidae, Hybotidae and Empididae (plus Microphorinae) and by Kahanpää and Grichanov (2004) for Dolichopodidae. Two updates to the dolichopodid checklist have since been published (Kahanpää 2006, 2013).

**Table 1.** Number of species by family.

Family	Number of species in			Level of knowledge
	World (Pape et al. 2011)	Europe	Finland	
Atelestidae	11	4	2	average
Brachystomatidae	147	13	4	average
Dolichopodidae	7235	790	260	good
Hybotidae	1972	442	143–144	average
Empididae	3049	803	175	average

## Checklist

suborder Brachycera Macquart, 1834

clade Eremoneura Lameere, 1906

superfamily Empidoidea Latreille, 1809

Uncertain family position within Empidoidea (*incertae familiae*)

***ITEAPHILA*** Zetterstedt, 1838

*Iteaphila macquarti* Zetterstedt, 1838

*Iteaphila nitidula* Zetterstedt, 1838

= *obscura* Zetterstedt, 1849

***ANTHEPISCOPUS*** Becker, 1891

*Anthepiscopus oedalinus* (Zetterstedt, 1838)

**EMPIDIDAE** Latreille, 1809

HEMERODROMIINAE Schiner, 1862

Chelipodini Hendel, 1936

***CHELIPODA*** Macquart, 1823

*Chelipoda albiseta* (Zetterstedt, 1838)

*Chelipoda inexspectata* Tuomikoski, 1966

*Chelipoda vocatoria* (Fallén, 1816)

***PHYLLODROMIA*** Zetterstedt, 1837

*Phyllodromia melanocephala* (Fabricius, 1794)

Hemerodromiini Schiner, 1862

***CHELIFERA*** Macquart, 1823

*Chelifera concinnicauda* Collin, 1927

= *lapponica* Frey, 1950

= *stigmatica* misid.

*Chelifera flavella* (Zetterstedt, 1838)

*Chelifera frigelii* (Zetterstedt, 1838)

*Chelifera precabunda* Collin, 1961

*Chelifera precatoria* (Fallén, 1816)

*Chelifera subangusta* Collin, 1961

*Chelifera trapezina* (Zetterstedt, 1838)

***HEMERODROMIA*** Meigen, 1822

*Hemerodromia adulatoria* Collin, 1927

*Hemerodromia oratoria* (Fallén, 1816)

*Hemerodromia raptoria* Meigen, 1830

EMPIDINAE Latreille, 1809

Empidini Latreille, 1809

***EMPIS*** Linnaeus, 1758

**sg. *Anacrostichus*** Bezzi, 1909

*Empis lucida* Zetterstedt, 1838

**sg. *Coptophlebia*** Bezzi, 1909

*Empis hyalipennis* Fallén, 1816

**sg. *Empis*** Linnaeus, 1758

*Empis acinerea* Chvala, 1985

= *cinerea* Zetterstedt, 1855 preocc.

*Empis bicuspidata* Collin, 1927

*Empis caudatula* Loew, 1867

*Empis chioptera* Meigen, 1804

*Empis laminata* Collin, 1927

*Empis nigripes* Fabricius, 1794

= *pennaria* Fallén, 1816

= *vernalis* Meigen, 1822

*Empis pennipes* Linnaeus, 1758

*Empis prodromus* Loew, 1867

*Empis staegeri* Collin, 1963

= *planetica* misid.

*Empis syrovatkai* Chvála, 1985

= *plumipes* Zetterstedt, 1842 preocc.

**sg. *Euempis*** Frey, 1953

*Empis picipes* Meigen, 1804

= *maculipes* Zetterstedt, 1842

*Empis tessellata* Fabricius, 1794

**sg. *Kritempis*** Collin, 1926

*Empis livida* Linnaeus, 1758

**sg. *Leptempis*** Collin, 1926

*Empis grisea* Fallén, 1816

**sg. *Platyptera*** Meigen, 1803

*Empis borealis* Linnaeus, 1758

**sg. *Xanthempis*** Bezzi, 1909

*Empis aemula* Loew, 1873

*Empis digramma* Meigen in Gistl, 1835

= *diagramma* misspelling

*Empis laetabilis* Collin, 1926

*Empis stercorea* Linnaeus, 1761

*Empis univittata* Loew, 1867

***RHAMPHOMYIA*** Meigen, 1822

**sg. *Aclonempis*** Collin, 1926

*Rhamphomyia galactoptera* Strobl, 1893

**sg. *Amydroneura*** Collin, 1926

*Rhamphomyia gibba* (Fallén, 1816)

**sg. *Holoclera*** Schiner, 1860

*Rhamphomyia bohemica* Barták & Kubík, 2012

= *caliginosa* misid.

= *trigemina* misid.

*Rhamphomyia culicina* (Fallén, 1816)

*Rhamphomyia nigripennis* (Fabricius, 1794)

*Rhamphomyia sciarina* (Fallén, 1816)

*Rhamphomyia umbripennis* Meigen, 1822

*Rhamphomyia variabilis* (Fallén, 1816)

= *tenuirostris* (Fallén, 1816)

**sg. *Lundstroemiella*** Frey, 1922

*Rhamphomyia dudai* Oldenberg, 1927

*Rhamphomyia hybotina* Zetterstedt, 1838

**sg. *Megacyttarus*** Bigot, 1880

*Rhamphomyia anomalina* Zetterstedt, 1838

*Rhamphomyia anomalipennis* Meigen, 1822

= *anomala* misid.

*Rhamphomyia crassirostris* (Fallén, 1816)

= *nigripes* auct. nec (Fabricius, 1794)

*Rhamphomyia gufitar* Frey, 1922

*Rhamphomyia maculipennis* Zetterstedt, 1842

= *tephraea* misid.

= *poissoni* misid.

*Rhamphomyia nodipes* (Fallén, 1816)

= *spissirostris* (Fallén, 1816)

*Rhamphomyia paradoxa* Wahlberg, 1844

**sg. *Pararhamphomyia*** Frey, 1922

*Rhamphomyia albidiventris* Strobl, 1898

= *woldstedti* Frey, 1913

*Rhamphomyia albipennis* (Fallén, 1816)

*Rhamphomyia albitarsis* Collin, 1926

*Rhamphomyia albissima* Frey, 1913

*Rhamphomyia alpina* Zetterstedt, 1838

*Rhamphomyia amoena* Loew, 1840

*Rhamphomyia angulifera* Frey, 1913

*Rhamphomyia breviventris* Frey, 1913

*Rhamphomyia caesia* Meigen, 1822

= *filata* Zetterstedt, 1842

*Rhamphomyia caudata* Zetterstedt, 1838

= *aethiops* Zetterstedt, 1838

*Rhamphomyia chibinensis* Frey, 1922

*Rhamphomyia cribrata* Oldenberg, 1927

*Rhamphomyia curvula* Frey, 1913

*Rhamphomyia dentata* Oldenberg, 1910

*Rhamphomyia fascipennis* Zetterstedt, 1838

*Rhamphomyia filicaudula* Frey, 1949

*Rhamphomyia fuscipennis* Zetterstedt, 1838

*Rhamphomyia fuscula* Zetterstedt, 1838

*Rhamphomyia geniculata* Meigen, 1830

= *plumipes* auct. nec (Meigen, 1804)

*Rhamphomyia griseola* (Zetterstedt, 1838)

= *dispar* Zetterstedt, 1838

= *aperta* Zetterstedt, 1859

*Rhamphomyia helleni* Frey, 1922

*Rhamphomyia lividiventris* Zetterstedt, 1838

*Rhamphomyia longestylata* Frey, 1916

= *caudata* auct. nec Zetterstedt, 1838

*Rhamphomyia lucidula* Zetterstedt, 1842

*Rhamphomyia marginata* (Fabricius, 1787)

*Rhamphomyia modesta* Wahlberg, 1844

*Rhamphomyia murina* Collin, 1926

*Rhamphomyia niveipennis* Zetterstedt, 1838

*Rhamphomyia obscura* Zetterstedt, 1838

*Rhamphomyia obscuripennis* Meigen, 1830

= *nitidicollis* Frey, 1913

*Rhamphomyia physoprocta* Frey, 1913

*Rhamphomyia pilifer* Meigen, 1838

= *dentipes* Zetterstedt, 1842

= *glaucella* Zetterstedt, 1842

= *intermedia* Frey, 1922

*Rhamphomyia plumifera* Zetterstedt, 1838

*Rhamphomyia poplitea* Wahlberg, 1844

*Rhamphomyia praestans* Frey, 1913

*Rhamphomyia pusilla* Zetterstedt, 1838

*Rhamphomyia rufipes* Zetterstedt, 1838

= *lapponica* Frey, 1955

*Rhamphomyia simplex* Zetterstedt, 1849

*Rhamphomyia subglaucella* Frey, 1922

*Rhamphomyia tibiella* Zetterstedt, 1842

*Rhamphomyia tipularia* (Fallén, 1816)

*Rhamphomyia unguiculata* Frey, 1913

Rhamphomyia sp. aff. albipennis

**sg. *Rhamphomyia*** Meigen, 1822

*Rhamphomyia albosegmentata* Zetterstedt, 1838

*Rhamphomyia cinerascens* (Meigen, 1804)

*Rhamphomyia coracina* Zetterstedt, 1849

*Rhamphomyia dorsata* Becker, 1915

= *phanerostigma* Frey, 1918

*Rhamphomyia hambergi* Frey, 1916

*Rhamphomyia ignobilis* Zetterstedt, 1859

= *attenuata* Frey, 1913

*Rhamphomyia laevipes* (Fallén, 1816)

= *tephraea* Meigen, 1822

*Rhamphomyia latifrons* Frey, 1913

*Rhamphomyia morio* Zetterstedt, 1838

*Rhamphomyia nitidula* Zetterstedt, 1842

*Rhamphomyia palmeni* Frey, 1913

*Rhamphomyia plumipes* (Meigen, 1804)

= *vespertilio* Zetterstedt, 1842

*Rhamphomyia reflexa* Zetterstedt, 1838

*Rhamphomyia spinipes* (Fallén, 1816)

*Rhamphomyia stigmosa* Macquart, 1827

= *conformis* auct. nec Kowarz, 1867

*Rhamphomyia sulcata* (Meigen, 1804)

= *tibialis* auct. nec Meigen, 1822

*Rhamphomyia trilineata* Zetterstedt, 1859

= *sulcatina* Collin, 1926

= *tibialis* auct. nec Meigen, 1822

= *propinqua* misid.

*Rhamphomyia vesiculosa* (Fallén, 1816)

Hilarini Collin, 1961

***HILARA*** Meigen, 1822

*Hilara abdominalis* Zetterstedt, 1838

= *obscuritarsis* Zetterstedt, 1859

*Hilara albitarsis* von Roser, 1840

*Hilara anglodanica* Lundbeck, 1913

*Hilara barbipes* Frey, 1908

*Hilara beckeri* Strobl, 1892

*Hilara biseta* Collin, 1927

*Hilara bistriata* Zetterstedt, 1842

*Hilara brevistyla* Collin, 1927

*Hilara campinosensis* Niesiolowski, 1986

*Hilara canescens* Zetterstedt, 1849

*Hilara chorica* (Fallén, 1816)

*Hilara clavipes* (Harris, 1776)

= *spinimana* Zetterstedt, 1838

*Hilara clypeata* Meigen, 1822

*Hilara coracina* Oldenberg, 1916

= *quadrifaria* Strobl, 1892

*Hilara cornicula* Loew, 1873

*Hilara discoidalis* Lundbeck, 1910

*Hilara diversipes* Strobl, 1892

= *germanica* Engel, 1941

*Hilara eviana* Straka, 1976

*Hilara femorella* Zetterstedt, 1842

*Hilara gallica* (Meigen, 1804)

*Hilara griseola* Zetterstedt, 1838

*Hilara hirta* Strobl, 1892

= *hirtella* misid.

*Hilara hybrida* Collin, 1961

*Hilara hyposeta* Straka, 1976

*Hilara implicata* Collin, 1927

*Hilara intermedia* (Fallén, 1816)

= *pubipes* Loew, 1873

*Hilara interstincta* (Fallén, 1816)

*Hilara monedula* Collin, 1927

= *longifurca* auct. nec Strobl, 1892

*Hilara lapponica* Chvála, 2002

*Hilara litorea* (Fallén, 1816)

*Hilara longivittata* Zetterstedt, 1842

*Hilara lurida* (Fallén, 1816)

*Hilara nigritarsis* Zetterstedt, 1838

= *infans* Zetterstedt, 1842

*Hilara nitidula* Zetterstedt, 1838

*Hilara pilipes* Zetterstedt, 1838

*Hilara pseudochorica* Strobl, 1892

*Hilara pulchripes* Frey, 1913

*Hilara quadrifasciata* Chvála, 2002

= *quadrivittata* auct. nec Meigen, 1822

*Hilara sturmii* Meigen, 1822

= *cingulata* misid.

*Hilara submaura* Collin, 1927

*Hilara tanythrix* Frey, 1913

*Hilara tenuinervis* Zetterstedt, 1838

*Hilara woodiella* Chvála, 1999

CLINOCERINAE Collin, 1928

***CLINOCERA*** Meigen, 1803

*Clinocera appendiculata* (Zetterstedt, 1838)

= *simplicinervis* Frey, 1913

*Clinocera aucta* (Zetterstedt, 1849)

*Clinocera nivalis* (Zetterstedt, 1838)

*Clinocera stagnalis* (Haliday, 1833)

*Clinocera wesmaeli* (Macquart, 1835)

***DOLICHOCEPHALA*** Macquart, 1823

*Dolichocephala guttata* (Haliday, 1833)

*Dolichocephala irrorata* (Fallén, 1815)

*Dolichocephala thomasi* Wagner, 1983

= *ocellata* misid.

***WIEDEMANNIA*** Zetterstedt, 1838

**sg. *Eucelidia*** Mik, 1881

*Wiedemannia zetterstedti* (Fallén, 1826)

**sg. *Philolutra*** Mik, 1881

*Wiedemannia bohemani* (Zetterstedt, 1838)

*Wiedemannia simplex* (Loew, 1862)

= *fallaciosa* auct. nec Loew, 1873

**sg. *Wiedemannia*** Zetterstedt, 1838

*Wiedemannia bistigma* (Curtis, 1834)

Uncertain subfamily position within Empididae (*incertae sedis*)

***HORMOPEZA*** Zetterstedt, 1838

*Hormopeza copulifera* Melander, 1927

*Hormopeza obliterata* Zetterstedt, 1838

***RAGAS*** Walker, 1837

*Ragas unica* Walker, 1837

**ATELESTIDAE** Hennig, 1970

ATELESTINAE Hennig, 1970

***ATELESTUS*** Walker, 1837

*Atelestus pulicarius* (Fallén, 1816)

***MEGHYPERUS*** Loew, 1850

*Meghyperus sudeticus* Loew, 1850

**HYBOTIDAE** Meigen, 1820

TRICHININAE Chvála, 1983

***TRICHINA*** Meigen, 1830

*Trichina bilobata* Collin, 1926

*Trichina clavipes* Meigen, 1830

*Trichina elongata* Haliday, 1833

*Trichina opaca* Loew, 1864

= *picipes* Tuomikoski, 1935

*Trichina pallipes* (Zetterstedt, 1838)

***TRICHINOMYIA*** Tuomikoski, 1959

*Trichinomyia flavipes* (Meigen, 1830)

*Trichinomyia fuscipes* (Zetterstedt, 1838)

OCYDROMIINAE Schiner, 1862

Ocydromiini Schiner, 1862

***CHVALAEA*** Papp & Földvári, 2001

*Chvalaea sopianae* Papp & Földvári, 2001

***LEPTODROMIELLA*** Tuomikoski, 1936

*Leptodromiella crassiseta* Tuomikoski, 1936

***LEPTOPEZA*** Macquart, 1834

*Leptopeza borealis* Zetterstedt, 1842

*Leptopeza flavipes* (Meigen, 1820)

***OCYDROMIA*** Meigen, 1820

*Ocydromia glabricula* (Fallén, 1816)

*Ocydromia melanopleura* Loew, 1840

OEDALEINAE Chvála, 1983

***ALLANTHALIA*** Melander, 1927

*Allanthalia pallida* (Zetterstedt, 1838)

***ANTHALIA*** Zetterstedt, 1838

*Anthalia schoenherri* Zetterstedt, 1838

***EUTHYNEURA*** Macquart, 1836

*Euthyneura albipennis* (Zetterstedt, 1842)

*Euthyneura gyllenhali* (Zetterstedt, 1838)

*Euthyneura myrtilli* Macquart, 1836

= *myricae* Haliday, 1851 misid.

***OEDALEA*** Meigen, 1820

*Oedalea freyi* Chvála, 1983

*Oedalea holmgreni* Zetterstedt, 1852

*Oedalea hybotina* (Fallén, 1816)

*Oedalea stigmatella* Zetterstedt, 1842

*Oedalea tibialis* Macquart, 1827

*Oedalea zetterstedti* Collin, 1926

TACHYDROMIINAE Meigen, 1822

Symballophthalmini Sinclair & Cumming, 2006

***SYMBALLOPHTHALMUS*** Mecker, 1889

*Symballophthalmus dissimilis* (Fallén, 1815)

*Symballophthalmus fuscitarsis* (Zetterstedt, 1859)

= *scapularis* Collin, 1961

Tachydromiini Meigen, 1822

***PLATYPALPUS*** Macquart, 1827

*Platypalpus agilis* (Meigen, 1822)

*Platypalpus albicornis* (Zetterstedt, 1842)

*Platypalpus albiseta* (Panzer, 1806)

*Platypalpus albocapillatus* (Fallén, 1815)

*Platypalpus alpinus* Chvála, 1971

*Platypalpus alter* (Collin, 1961)

*Platypalpus annulatus* (Fallén, 1815)

= *fulvipes* (Meigen, 1822)

*Platypalpus annulipes* (Meigen, 1822)

*Platypalpus articulatoides* (Frey, 1918)

? *Platypalpus articulatus* Macquart, 1827

*Platypalpus ater* (Wahlberg, 1844)

= *atra* misspelling

*Platypalpus boreoalpinus* Frey, 1943

*Platypalpus brachystylus* (Bezzi, 1892)

= *brunneitibia* (Strobl, 1899)

*Platypalpus brevicornis* (Zetterstedt, 1842)

*Platypalpus calceatus* (Meigen, 1822)

*Platypalpus candicans* (Fallén, 1815)

*Platypalpus ciliaris* (Fallén, 1816)

*Platypalpus confiformis* Chvála, 1971

*Platypalpus confinis* (Zetterstedt, 1842)

*Platypalpus cothurnatus* Macquart, 1827

*Platypalpus cryptospina* (Frey, 1909)

= *tantulus* (Collin, 1961)

*Platypalpus cursitans* (Fabricius, 1775)

= *bicolor* (Meigen, 1804)

*Platypalpus ecalceatus* (Zetterstedt, 1838)

*Platypalpus excavatus* Yang & Yao, 2007

= *excisus* (Becker, 1907) preocc.

*Platypalpus exilis* (Meigen, 1822)

*Platypalpus fenestella* Kovalev, 1971

*Platypalpus flavicornis* (Meigen, 1822)

*Platypalpus fuscicornis* (Zetterstedt, 1842)

*Platypalpus hackmani* Chvála, 1972

*Platypalpus infectus* (Collin, 1926)

*Platypalpus interstinctus* (Collin, 1926)

*Platypalpus laestadianorum* (Frey, 1913)

*Platypalpus lapponicus* Frey, 1943

*Platypalpus longicornis* (Meigen, 1822)

*Platypalpus longiseta* (Zetterstedt, 1842)

= *extricatus* (Collin, 1926)

*Platypalpus luteicornis* (Meigen, 1838)

= *difficilis* (Frey, 1907)

= *interjectus* (Lundbeck, 1910)

*Platypalpus luteus* (Meigen, 1804)

*Platypalpus maculus* (Zetterstedt, 1842)

*Platypalpus maculimanus* (Zetterstedt, 1842)

= *articulatus* auct. nec Macquart, 1827

*Platypalpus maculipes* (Meigen, 1822)

*Platypalpus major* (Zetterstedt, 1842)

*Platypalpus melancholicus* (Collin, 1961)

*Platypalpus minutus* (Meigen, 1804)

*Platypalpus nigricoxa* (Mik, 1884)

*Platypalpus nigritarsis* (Fallén, 1816)

*Platypalpus nigrosetosus* (Strobl, 1893)

*Platypalpus nonstriatus* Strobl, 1901

*Platypalpus notatus* (Meigen, 1822)

*Platypalpus pallidicornis* (Collin, 1926)

*Platypalpus pallidicoxa* (Frey, 1913)

*Platypalpus pallidiventris* (Meigen, 1822)

= *flavipes* (Fabricius, 1794)

*Platypalpus pallipes* (Fallén, 1815)

*Platypalpus pectoralis* (Fallén, 1815)

*Platypalpus pseudofulvipes* (Frey, 1909)

= *coarctatus* (Collin, 1926)

*Platypalpus pseudorapidus* Kovalev, 1971

*Platypalpus pulicarius* (Meigen, 1830)

*Platypalpus rapidus* (Meigen, 1822)

*Platypalpus sahlbergi* (Frey, 1909)

*Platypalpus scandinavicus* Chvála, 1972

*Platypalpus sordidus* (Zetterstedt, 1838)

*Platypalpus stabilis* (Collin, 1961)

*Platypalpus stackelbergi* Kovalev, 1971

*Platypalpus stigmatellus* (Zetterstedt, 1842)

*Platypalpus strigifrons* (Zetterstedt, 1849)

*Platypalpus subbrevis* (Frey, 1913)

*Platypalpus subtilis* (Collin, 1926)

*Platypalpus sylvicola* (Collin, 1926)

*Platypalpus tuomikoskii* Chvála, 1972

*Platypalpus unguiculatus* (Zetterstedt, 1838)

*Platypalpus verralli* (Collin, 1926)

*Platypalpus vividus* (Meigen, 1838)

= *albisetoides* Chvála, 1973

*Platypalpus zetterstedti* Chvála, 1971

***TACHYDROMIA*** Meigen, 1803

*Tachydromia aemula* (Loew, 1864)

*Tachydromia arrogans* (Linnaeus, 1761)

*Tachydromia connexa* Meigen, 1822

*Tachydromia incompleta* (Becker, 1900)

*Tachydromia lundstroemi* (Frey, 1913)

*Tachydromia morio* (Zetterstedt, 1838)

*Tachydromia punctifera* (Becker, 1900)

*Tachydromia sabulosa* Meigen, 1830

*Tachydromia terricola* Zetterstedt, 1819

*Tachydromia umbrarum* Haliday, 1833

***TACHYPEZA*** Meigen, 1830

*Tachypeza fennica* Tuomikoski, 1932

*Tachypeza fuscipennis* (Fallén, 1815)

*Tachypeza heeri* (Zetterstedt, 1838)

*Tachypeza nubila* (Meigen, 1804)

*Tachypeza truncorum* (Fallén, 1815)

*Tachypeza winthemi* (Zetterstedt, 1838)

Drapetini Collin, 1961

***CHERSODROMIA*** Haliday, 1851

*Chersodromia arenaria* (Haliday, 1833)

*Chersodromia cursitans* (Zetterstedt, 1819)

***CROSSOPALPUS*** Bigot, 1857

*Crossopalpus curvinervis* (Zetterstedt, 1842)

*Crossopalpus curvipes* (Meigen, 1822)

*Crossopalpus humilis* (Frey, 1913)

*Crossopalpus nigritellus* (Zetterstedt, 1842)

*Crossopalpus setiger* (Loew, 1859)

***DRAPETIS*** Meigen, 1822

*Drapetis arcuata* Loew, 1859

*Drapetis assimilis* (Fallén, 1815)

*Drapetis exilis* Meigen, 1822

*Drapetis infitialis* Collin, 1961

*Drapetis ingrica* Kovalev, 1972

*Drapetis parilis* Collin, 1926

*Drapetis pusilla* Loew, 1859

*Drapetis simulans* Collin, 1961

***Elaphropeza*** Macquart, 1827

*Elaphropeza ephippiata* (Fallén, 1815)

***STILPON*** Loew, 1859

*Stilpon graminum* (Fallén, 1815)

HYBOTINAE Meigen, 1820

Bicellariini Sinclair & Cumming, 2006

***BICELLARIA*** Macquart, 1823

= ***Cyrtoma*** Meigen, 1824

*Bicellaria austriaca* Tuomikoski, 1955

*Bicellaria intermedia* Lundbeck, 1910

*Bicellaria nigra* (Meigen, 1824)

*Bicellaria pilosa* Lundbeck, 1910

*Bicellaria simplicipes* (Zetterstedt, 1842)

*Bicellaria spuria* (Fallén, 1816)

*Bicellaria subpilosa* Collin, 1926

*Bicellaria sulcata* (Zetterstedt, 1842)

*Bicellaria uvens* Melander, 1928

= *bisetosa* Tuomikoski, 1936

Hybotini Meigen, 1820

***HYBOS*** Meigen, 1803

*Hybos culiciformis* (Fabricius, 1775)

*Hybos femoratus* (Müller, 1776)

= *femoralis* misspelling

*Hybos grossipes* (Linnaeus, 1767)

***SYNDYAS*** Loew, 1857

*Syndyas nigripes* (Zetterstedt, 1842)

**BRACHYSTOMATIDAE** Melander, 1908

TRICHOPEZINAE Vaillant, 1981

***GLOMA*** Meigen, 1822

*Gloma fuscipennis* Meigen, 1822

***HELEODROMIA*** Haliday, 1833

**sg. *Heleodromia*** Haliday, 1833

*Heleodromia immaculata* Haliday, 1833

***TRICHOPEZA*** Rondani, 1856

*Trichopeza albocincta* (Boheman, 1863)

= *albicincta* Frey, 1913

*Trichopeza longicornis* (Meigen, 1822)

**DOLICHOPODIDAE** Latreille, 1809

MICROPHORINAE Collin, 1960

***MICROPHOR*** Macquart, 1827

= ***Microphorus*** misspelling

*Microphor anomalus* (Meigen, 1824)

*Microphor crassipes* Macquart, 1827

*Microphor holosericeus* (Meigen, 1804)

= *velutinus* Macquart, 1827

PARATHALASSIINAE Chvála, 1981

***MICROPHORELLA*** Becker, 1909

*Microphorella praecox* (Loew, 1864)

DOLICHOPODINAE Latreille, 1809

***DOLICHOPUS*** Latreille, 1796

= ***Hygroceleuthus*** Loew, 1857

**sg. *Dolichopus*** Latreille, 1796

*Dolichopus acuticornis* Wiedemann, 1817

*Dolichopus annulitarsis* Ringdahl, 1920

*Dolichopus apicalis* Zetterstedt, 1849

*Dolichopus argyrotarsis* Wahlberg, 1850

*Dolichopus armillatus* Wahlberg, 1850

= *stenhammari* var. b Zetterstedt, 1843

*Dolichopus atripes* Meigen, 1824

*Dolichopus austriacus* Parent, 1927

*Dolichopus bonsdorffi* Frey, 1915

*Dolichopus brevipennis* Meigen, 1824

*Dolichopus caligatus* Wahlberg, 1850

= *flavipes* misid.

= *albifrons* misid.

*Dolichopus calinotus* Loew, 1871

*Dolichopus campestris* Meigen, 1824

*Dolichopus cilifemoratus* Macquart, 1827

= *pseudocilifemoratus* Stackelberg, 1930

*Dolichopus cinctipes* Wahlberg, 1850

*Dolichopus claviger* Stannius, 1831

*Dolichopus clavipes* Haliday, 1832

*Dolichopus costalis* Frey, 1915

*Dolichopus discifer* Stannius, 1831

?= *nigricornis* Meigen, 1824

*Dolichopus discimanus* Wahlberg, 1851

*Dolichopus fraterculus* Zetterstedt, 1843

*Dolichopus griseipennis* Stannius, 1831

*Dolichopus gubernator* Mik, 1878

*Dolichopus hilaris* Loew, 1862

*Dolichopus lancearius* Hedström, 1966

*Dolichopus latilimbatus* Macquart, 1827

*Dolichopus latipennis* Fallén, 1823

*Dolichopus lepidus* Staeger, 1842

= *cruralis* Wahlberg, 1850

= *lapponicus* Becker, 1917

*Dolichopus linearis* Meigen, 1824

*Dolichopus lineatocornis* Zetterstedt, 1843

*Dolichopus longicornis* Stannius, 1831

*Dolichopus longitarsis* Stannius, 1831

*Dolichopus maculipennis* Zetterstedt, 1843

*Dolichopus mannerheimi* Zetterstedt, 1838

*Dolichopus migrans* Zetterstedt, 1843

*Dolichopus nigripes* Fallén, 1823

*Dolichopus nitidus* Fallén, 1823

*Dolichopus notatus* Staeger, 1842

= *notabilis* Zetterstedt, 1843

*Dolichopus nubilus* Meigen, 1824

*Dolichopus pennatus* Meigen, 1824

*Dolichopus picipes* Meigen, 1824

= *consimilis* Wahlberg, 1850

*Dolichopus planitarsis* Fallén, 1823

*Dolichopus plumipes* (Scopoli, 1763)

= *parvicaudatus* Zetterstedt, 1843

= *pectinitarsis* Stenhammar, 1851

*Dolichopus popularis* Wiedemann, 1817

*Dolichopus pseudomigrans* Ringdahl, 1928

*Dolichopus punctum* Meigen, 1824

*Dolichopus remipes* Wahlberg, 1839

*Dolichopus rupestris* Haliday, 1833

*Dolichopus ruthei* Loew, 1847

*Dolichopus sabinus* Haliday, 1838

*Dolichopus setiger* Negrobov, 1973

*Dolichopus signatus* Meigen, 1824

*Dolichopus signifer* Haliday, 1832

*Dolichopus simplex* Meigen, 1824

*Dolichopus stenhammari* Zetterstedt, 1843

= *annulipes* Zetterstedt, 1838 invalidated

*Dolichopus subpennatus* d’Assis-Fonseca, 1976

*Dolichopus trivialis* Haliday, 1832

= *cilifemoratus* auct. nec Macquart, 1827

*Dolichopus ungulatus* (Linnaeus, 1758)

*Dolichopus urbanus* Meigen, 1824

*Dolichopus vitripennis* Meigen, 1824

*Dolichopus wahlbergi* Zetterstedt, 1843

*Dolichopus zetterstedti* Stenhammar, 1852

**sg. *Macrodolichopus*** Stackelberg, 1933

*Dolichopus diadema* Haliday, 1832

***ETHIROMYIA*** Brooks, 2005

*Ethiromyia chalybeus* (Wiedemann, 1817)

***GYMNOPTERNUS*** Loew, 1857

*Gymnopternus aerosus* (Fallén, 1823)

*Gymnopternus angustifrons* (Staeger, 1842)

*Gymnopternus brevicornis* (Staeger, 1842)

*Gymnopternus celer* (Meigen, 1824)

*Gymnopternus metallicus* (Stannius, 1831)

***HERCOSTOMUS*** Loew, 1857

*Hercostomus germanus* (Wiedemann, 1817)

*Hercostomus nigrilamellatus* (Macquart, 1827)

*Hercostomus nigriplantis* (Stannius, 1831)

*Hercostomus sahlbergi* (Zetterstedt, 1838)

***SYBISTROMA*** Meigen, 1824

*Sybistroma discipes* (Germar, 1817)

*Sybistroma obscurellum* (Fallén, 1823)

***TACHYTRECHUS*** Haliday, 1851

= ***Ammobates*** Stannius, 18312 preocc.

*Tachytrechus ammobates* (Haliday, 1851)

= *plumipes* (Fallén, 1823) preocc.

*Tachytrechus hamatus* Loew, 1871

*Tachytrechus notatus* (Stannius, 1831)

SCIAPODINAE Becker, 1917

***SCIAPUS*** Zeller, 1842

= ***Sciopus*** misspelling

*Sciapus albifrons* (Meigen, 1830)

*Sciapus basilicus* Meuffels & Grootaert, 1990

*Sciapus lobipes* (Meigen, 1824)

*Sciapus longulus* (Fallén, 1823)

*Sciapus maritimus* Becker, 1918

= *flavomaculatus* Ringdahl, 1949

*Sciapus platypterus* (Fabricius, 1805)

*Sciapus wiedemanni* (Fallén, 1823)

*Sciapus zonatulus* (Zetterstedt, 1843)

= *contristans* misid.

SYMPYCNINAE Aldrich, 1905

***CAMPSICNEMUS*** Haliday, 1851

= ***Ectomus*** Mik, 1878

*Campsicnemus alpinus* (Haliday, 1833)

*Campsicnemus armatus* (Zetterstedt, 1849)

*Campsicnemus articulatellus* (Zetterstedt, 1843)

= *pilosellus* (Zetterstedt, 1843)

= *dasycnemus* Loew, 1857

*Campsicnemus compeditus* Loew, 1857

*Campsicnemus curvipes* (Fallén, 1823)

*Campsicnemus femoratus* Ringdahl, 1949

*Campsicnemus loripes* (Haliday, 1832)

*Campsicnemus lumbatus* Loew, 1857

*Campsicnemus marginatus* Loew, 1857

*Campsicnemus paradoxus* (Wahlberg, 1844)

*Campsicnemus picticornis* (Zetterstedt, 1843)

*Campsicnemus pumilio* (Zetterstedt, 1843)

= *pectinulatus* Loew, 1864

*Campsicnemus pusillus* (Meigen, 1824)

*Campsicnemus scambus* (Fallén, 1823)

***LAMPROCHROMUS*** Mik, 1878

*Lamprochromus strobli* Parent, 1925

***SYMPYCNUS*** Loew, 1857

*Sympycnus aeneicoxa* (Meigen, 1824)

*Sympycnus pulicarius* (Fallén, 1823)

?= *annulipes* (Meigen, 1824)

?= *desoutteri* Parent, 1925

***SYNTORMON*** Loew, 1857

= ***Bathycranium*** Strobl, 1892

*Syntormon bicolorellus* (Zetterstedt, 1843)

*Syntormon filiger* Verrall, 1912

= *rufipes* misid.

= *simplicipes* Frey, 1915

*Syntormon freymuthae* Loew, 1873

= *Syntormon denticulatus* misid.

*Syntormon metathesis* (Loew, 1850)

*Syntormon pallipes* (Fabricius, 1794)

*Syntormon pumilus* (Meigen, 1824)

*Syntormon tarsatus* (Fallén, 1823)

= *aulicus* misid.

***TELMATURGUS*** Mik, 1874

*Telmaturgus tumidulus* (Raddatz, 1873)

***TEUCHOPHORUS*** Loew, 1857

= ***Teucophorus*** misspelling

*Teuchophorus monacanthus* Loew, 1859

*Teuchophorus nigricosta* (von Roser, 1840)

= *pectinifer* Kowarz, 1868

= *signatus* Zetterstedt, 1849

*Teuchophorus spinigerellus* (Zetterstedt, 1843)

DIAPHORINAE Schiner, 1864

***ARGYRA*** Macquart, 1834

= ***Leucostola*** Loew, 1857

*Argyra argentina* (Meigen, 1824)

*Argyra argyria* (Meigen, 1824)

*Argyra auricollis* (Meigen, 1824)

*Argyra diaphana* (Fabricius, 1775)

*Argyra elongata* (Zetterstedt, 1843)

*Argyra ilonae* Geoffries, 1989

= *confinis* (Zetterstedt, 1849) preocc.

*Argyra leucocephala* (Meigen, 1824)

*Argyra magnicornis* (Zetterstedt, 1838)

*Argyra setulipes* Becker, 1918

*Argyra setimana* Loew, 1859

= *subarctica* misid.

*Argyra spoliata* Kowarz, 1879

*Argyra vestita* (Wiedemann, 1817)

***ASYNDETUS*** Loew, 1869

*Asyndetus latifrons* (Loew, 1857)

***CHRYSOTUS*** Meigen, 1824

*Chrysotus angulicornis* Kowarz, 1874

*Chrysotus cilipes* Meigen, 1824

*Chrysotus cupreus* (Macquart, 1827)

*Chrysotus femoratus* Zetterstedt, 1843

*Chrysotus gramineus* (Fallén, 1823)

= *microcerus* Kowarz, 1874

= *varians* Kowarz, 1874

*Chrysotus laesus* (Wiedemann, 1817)

= *amplicornis* Zetterstedt, 1849

*Chrysotus neglectus* (Wiedemann, 1817)

*Chrysotus obscuripes* Zetterstedt, 1838

= *kowarzi* Lundbeck, 1912

*Chrysotus pulchellus* Kowarz, 1874

*Chrysotus suavis* Loew, 1857

***DIAPHORUS*** Meigen, 1824

*Diaphorus hoffmannseggi* Meigen, 1830

*Diaphorus nigricans* Meigen, 1824

*Diaphorus oculatus* (Fallén, 1823)

***MELANOSTOLUS*** Kowarz, 1884

*Melanostolus melancholicus* (Loew, 1869)

MEDETERINAE Lioy, 1864

***DOLICHOPHORUS*** Lichtward, 1902

*Dolichophorus kerteszi* Lichtward, 1902

***MEDETERA*** Fischer von Waldheim, 1819

*Medetera abstrusa* Thuneberg, 1955

*Medetera acanthura* Negrobov & Thuneberg, 1970

*Medetera adjaniae* Gosseries, 1989

= *breviseta* Parent, 1927 preocc.

*Medetera ambigua* (Zetterstedt, 1843)

*Medetera apicalis* (Zetterstedt, 1843)

*Medetera belgica* Parent, 1936 *sensu* Negrobov & Stackelberg, 1972

*Medetera betulae* Ringdahl, 1949

*Medetera borealis* Thuneberg, 1955

*Medetera cuspidata* Collin, 1941

*Medetera dichrocera* Kowarz, 1877

*Medetera excellens* Frey, 1909

*Medetera fasciata* Frey, 1915

*Medetera freyi* Thuneberg, 1955

*Medetera fumida* Negrobov, 1967

*Medetera impigra* Collin, 1941

*Medetera incrassata* Frey, 1909

*Medetera infumata* Loew, 1857

*Medetera inspissata* Collin, 1952

*Medetera jacula* (Fallén, 1823)

*Medetera jugalis* Collin, 1941

*Medetera melancholica* Lundbeck, 1912

*Medetera muralis* Meigen, 1824

*Medetera nitida* (Macquart, 1834)

= *stackelbergi* Parent, 1927

*Medetera obscura* (Zetterstedt, 1838)

*Medetera pallipes* (Zetterstedt, 1843)

*Medetera parenti* Stackelberg, 1925

= *collini* Thuneberg, 1955

*Medetera pinicola* Kowarz, 1877

= *nuortevai* Thuneberg, 1955

*Medetera plumbella* Meigen, 1824

*Medetera prjachinae* Negrobov & Stackelberg, 1974

*Medetera protuberans* Negrobov, 1967

*Medetera pseudoapicalis* Thuneberg, 1955

*Medetera seguyi* Parent, 1926

*Medetera senicula* Kowarz, 1877

*Medetera setiventris* Thuneberg, 1955

*Medetera signaticornis* Loew, 1857

*Medetera striata* Parent, 1927

*Medetera tristis* (Zetterstedt, 1838)

*Medetera vagans* Becker, 1917

= *fennica* Thuneberg, 1955

*Medetera veles* Loew, 1861

= *bilineata* Frey, 1915

*Medetera zinovjevi* Negrobov, 1967

***SYSTENUS*** Loew, 1857

*Systenus bipartitus* (Loew, 1850)

*Systenus pallipes* (von Roser, 1840)

= *adpropinquus* (Loew, 1857)

*Systenus scholtzi* (Loew, 1850)

***THRYPTICUS*** Gerstäcker, 1864

*Thrypticus atomus* Frey, 1915

*Thrypticus bellus* Loew, 1869

*Thrypticus cuneatus* (Becker, 1917)

*Thrypticus divisus* Strobl, 1880

= *fennicus* Becker, 1917

*Thrypticus intercedens* Negrobov, 1967

*Thrypticus laetus* Verrall, 1912

*Thrypticus nigricauda* Wood, 1913

*Thrypticus pollinosus* Verrall, 1912

*Thrypticus pruinosus* Parent, 1932

*Thrypticus smaragdinus* Gerstäcker, 1864

*Thrypticus tarsalis* Parent, 1932

*Thrypticus* sp. A

HYDROPHORINAE Lioy, 1864

***HYDROPHORUS*** Fallén, 1823

*Hydrophorus albiceps* Frey, 1915

*Hydrophorus alpinus* Wahlberg, 1844

*Hydrophorus altivagus* Aldrich, 1911

= *wahlgreni* Frey, 1915

*Hydrophorus bipunctatus* (Lehmann, 1822)

*Hydrophorus borealis* Loew, 1857

*Hydrophorus brunnicosus* Loew, 1857

*Hydrophorus callosoma* Frey, 1915

= *albosignatus* Ringdahl, 1919

*Hydrophorus freyi* Storå, 1954

*Hydrophorus geminus* Frey, 1915

*Hydrophorus litoreus* Fallén, 1823

*Hydrophorus nebulosus* Fallén, 1823

*Hydrophorus norvegicus* Ringdahl, 1928

*Hydrophorus pectinatus* Gerstäcker, 1864

= *forcipatus* Frey, 1915

*Hydrophorus pilipes* Frey, 1915

*Hydrophorus praecox* (Lehmann, 1822)

*Hydrophorus rufibarbis* Gerstäcker, 1864

= *micans* Frey, 1915

*Hydrophorus signiferus* Coquillett, 1899

= *magnicornis* Frey, 1915

*Hydrophorus viridis* (Meigen, 1824)

***LIANCALUS*** Loew, 1857

*Liancalus virens* (Scopoli, 1763)

***PEODES*** Loew, 1857

*Peodes petsamoensis* Frey, 1930

***SCELLUS*** Loew, 1857

*Scellus spinimanus* (Zetterstedt, 1843)

***THINOPHILUS*** Wahlberg, 1844

**sg. *Thinophilus*** Wahlberg, 1844

*Thinophilus flavipalpis* (Zetterstedt, 1843)

*Thinophilus ruficornis* (Haliday, 1838)

NEURIGONINAE Aldrich, 1905

***NEURIGONA*** Rondani, 1856

*Neurigona abdominalis* (Fallén, 1823)

*Neurigona pallida* (Fallén, 1823)

*Neurigona quadrifasciata* (Fabricius, 1781)

*Neurigona suturalis* (Fallén, 1823)

*Neurigona* sp. A

RHAPHIINAE Bigot, 1852

***RHAPHIUM*** Meigen, 1803

= ***Xiphandrium*** Loew, 1857

= ***Porphyrops*** auctt.

*Rhaphium albifrons* (Zetterstedt, 1843)

*Rhaphium appendiculatum* (Zetterstedt, 1849)

= *macrocerum* auct. nec Meigen, 1824

*Rhaphium basale* Loew, 1850

*Rhaphium caliginosum* Meigen, 1824

= *zetterstedti* (Parent, 1925)

*Rhaphium commune* (Meigen, 1824)

= *spinicoxa* Loew, 1850

*Rhaphium confine* Zetterstedt, 1843

*Rhaphium crassipes* (Meigen, 1824)

*Rhaphium discolor* Zetterstedt, 1838

= *consobrinum* Zetterstedt, 1843

= *riparium* auct. nec (Meigen, 1824: 53)

*Rhaphium elegantulum* (Meigen, 1824)

*Rhaphium fasciatum* Meigen, 1824

*Rhaphium fascipes* (Meigen, 1824)

*Rhaphium fissum* Loew, 1850

*Rhaphium glaciale* (Ringdahl, 1920)

*Rhaphium holmgreni* (Mik, 1878)

= *luteipenne* (Frey, 1915)

*Rhaphium lanceolatum* Loew, 1850

= *caliginosum* auct. nec Meigen, 1824

*Rhaphium laticorne* (Fallén, 1823)

= *nemorum* Meigen, 1830

*Rhaphium latimanum* Kahanpää, 2007

*Rhaphium longicorne* (Fallén, 1823)

*Rhaphium micans* (Meigen, 1824)

*Rhaphium monotrichum* Loew, 1850

= *auctum* misid.

*Rhaphium nasutum* (Fallén, 1823)

*Rhaphium nigribarbatum* (Becker, 1900)

*Rhaphium patulum* (Raddatz, 1873)

= *antennatum* misid.

*Rhaphium penicillatum* Loew, 1850

*Rhaphium riparium* (Meigen, 1824)

= *praerosum* Loew, 1850

*Rhaphium rivale* (Loew, 1869)

*Rhaphium tridactylum* (Frey, 1915)

*Rhaphium umbripenne* (Frey, 1915)

ACHALCINAE Grootaert & Meuffels, 1997

***ACHALCUS*** Haliday, 1857

*Achalcus cinereus* (Haliday, 1851)

*Achalcus flavicollis* (Meigen, 1824)

*Achalcus nigropunctatus* Pollét & Brunhues, 1996

*Achalcus vaillanti* Brunhues, 1987

PELOROPEODINAE Robinson, 1970

***CHRYSOTIMUS*** Loew, 1857

*Chrysotimus molliculus* (Fallén, 1823)

***MICROMORPHUS*** Mik, 1878

*Micromorphus claripennis* (Strobl, 1899)

XANTHOCHLORINAE Aldrich, 1905

***XANTHOCHLORUS*** Loew, 1857

*Xanthochlorus ornatus* (Haliday, 1832)

*Xanthochlorus tenellus* (Wiedemann, 1817)

## Excluded species

*Chelifera astigma* Collin, 1927 misidentified

*Chrysotus arcticus* Frey, 1915 not found within present borders

*Chrysotus longipalpus* Aldrich, 1896 imported

= *pallidipalpus* van Duzee, 1933

*Chrysotus ringdahli* Parent, 1929 not found within present borders

*Clinocera nigra* Meigen, 1804 misidentified

*Crossopalpus abditus* Kovalev, 1972 not found within present borders

*Dolichopus angustipennis* Kertesz, 1901 not found within present borders

= *adustus* Frey, 1915

*Dolichopus grandicornis* Wahlberg, 1850 not found within present borders

*Dolichopus mediicornis* Verrall, 1875 not found within present borders

*Dolichopus plumitarsis* Fallén, 1823 misidentified

*Dolichopus propinquus* Zetterstedt, 1852 misidentified

*Drapetis incompleta* Collin, 1926 misidentified

*Empis punctata* Meigen, 1804 not found within present borders

*Gymnopternus assimilis* (Stæger, 1842) misidentified

*Hilara flavipes* Meigen, 1822 misidentified

*Hilara maura* (Fabricius, 1776) not found within present borders

*Hilara scrobiculata* Loew, 1873 misidentified

*Hilara tetragramma* Loew, 1873 misidentified

*Hydrophorus balticus* (Meigen, 1824) not found within present borders

*Hydrophorus ponojensis* Frey, 1915 not found within present borders

*Iteaphila furcata* (Zetterstedt, 1842) not found within present borders

*Medetera annulitarsus* von Roser, 1840 misidentified

*Medetera glauca* Loew, 1869 misidentified

*Medetera feminina* Negrobov, 1967 misidentified

*Medetera truncorum* Meigen, 1824 imported

*Oedalea flavipes* Zetterstedt, 1842 misidentified

*Parathalassius krogerusi* Frey, 1927 nomen nudum

*Rhamphomyia tibialis* Meigen, 1822 misidentified

*Rhaphium antennatum* (Carlier, 1835) misidentified

*Rhaphium obscuripes* Zetterstedt, 1849 misidentified

*Rhaphium suave* (Loew, 1859) not found within present borders

*Tachytrechus insignis* (Stannius, 1831) misidentified

*Trichoclinocera lapponica* (Ringdahl, 1933) not found within present borders

## Notes

***Dolichopus caligatus* Wahlberg, 1850** was synonymized with *Dolichopus flavipes* Stannius, 1831 by Becker (1918) but Collin (1940) reinstated *Dolichopus caligatus* as a valid species. True *Dolichopus flavipes* (if it is a valid species) has not been found in Finland but some specimens have been recorded from Northwestern European Russia.

***Dolichopus stenhammari* Zetterstedt, 1843**. Zetterstedt (1838) first described this species as *Dolichopus annulipes*. Later he proposed *Dolichopus stenhammari* Zett., 1843 as an (unnecessary) replacement name for the taxon: *Porphyrops annulipes* Meigen, 1824 was recognized in *Dolichopus* at the time, but only as a junior synonym of what is now *Sympycnus pulicarius* (Fallén, 1823) (Zetterstedt 1843). Nonetheless this act means, according to article 59.3 of The Code (ICZN 1999), that *Dolichopus annulipes* Zett., 1838 is permanently invalid.

***Hilara pseudochorica* Strobl, 1892**. Erroneously deleted from the Finnish checklist by Kahanpää and Winqvist (2005).

***Iteaphila furcata* (Zetterstedt 1842)**. Sinclair and Shamshev (2012) list a specimen from Kuusamo with code label 1546. According to R. Frey’s notes this specimen was collected in 1917 from eastern Kuusamo, an area ceded to Russia in 1944.

***Neurigona* sp. A** is probably *Neurigona uralensis* Becker, 1918.

***Platypalpus articulatus*** and ***Platypalpus maculimanus***. According to Allen (1986) these two names are not synonymous and the species identified as *Platypalpus articulatus* Macquart by Chvála (1975, 1989) is in fact *Platypalpus maculimanus* (Zett.). Upon re-examination, all Finnish material previously identified as *Platypalpus articulatus* belonged to *Platypalpus maculimanus* and *Platypalpus articulatoides* Frey. It remains unclear whether the real *Platypalpus articulatus* occurs in Finland.

**Rhamphomyia sp. nr. albipennis**. An apparently undescribed species near *Rhamphomyia albipennis* (Fallén, 1816) (K. Winqvist & M. Barták, priv. comm.).

***Sympycnus pulicarius* (Fallén, 1823)**. Pollet (2004) (Fauna Europaea) treats *Sympycnus annulipes* (Meigen, 1824) and *Sympycnus desoutteri* Parent, 1925 as valid species and lists *Sympycnus pulicarius* (Fallén, 1823) as a synonym of the latter but notes that it might actually be a synonym of *Sympycnus annulipes*.

***Thrypticus* sp. A** is probably *Thrypticus incanus* Negrobov, 1967 described from the Leningrad Oblast, Russia (Negrobov 1967).

***Trichoclinocera lapponica* (Ringdahl, 1933)**. The type locality of this species, ”Lappland nära Tjuonajaure”, (Ringdahl 1933) is in Sweden, not Finland as stated in Chvála and Wagner (1989).

## References

[B1] Allen AA (1986) *Platypalpus articulatoides* (Frey) (Dipt., Empididae) new to Britain. Entomologist’s Record and Journal of Variation 98: 177–179

[B2] Becker T (1918) Dipterologische Studien. Dolichopodidae. A. Paläarktische Region. Nova Acta Academiae Caesareae Leopoldinisch-Carolinae Germanicae Naturae Curiosorum 104: 35–214

[B3] Chvála M (1975) The Tachydromiinae (Dipt.Empididae) of Fennoscandia and Denmark. Fauna Entomologica Scandinavica 3. Scandinavian Science Press Ltd., Klampenborg, 336 pp

[B4] Chvála M (1983) The Empidoidea (Diptera) of Fennoscandia and Denmark. II. General Part. The Families Hybotidae, Atelestidae and Microphoridae. Fauna Entomologica Scandinavica 12. Scandinavian Science Press Ltd., Klampenborg, 279 pp

[B5] Chvála M (1989) Monograph of Northern and Central European species of *Platypalpus* (Diptera, Hybotidae), with data on the occurrence in Czechoslovakia. Acta Universitatis Carolinae–Biologica 32: 209–376

[B6] Chvála M Wagner R (1989) Family Empididae. In: Soós A Papp L (Eds) Catalogue of Palaearctic Diptera. Therevidae-Empididae. Volume 6. Akademiai Kiado, Budapest & Elsevier, Amsterdam, 228–336

[B7] Collin JE (1940) Critical notes on some recent synonymy affecting British species of Dolichopodidae (Diptera). Entomologist’s Monthly Magazine 76: 261–271

[B8] Kahanpää J (2006) First update to the checklist of Finnish Dolichopodidae (Diptera). International Journal of Dipterological Research 17(2): 121–125

[B9] Kahanpää J (2013) A second update to the checklist of Finnish long-legged flies (Diptera: Dolichopodidae), with a re-evaluation of the status of *Hydrophorus callosoma* Frey, 1915. Biodiversity Data Journal 1: . doi: 10.3897/BDJ.1.e976 10.3897/BDJ.1.e976 PMC3964712 24723770

[B10] Kahanpää J Grichanov IY (2004) A check-list of Finnish long-legged flies (Diptera: Dolichopodidae). International Journal of Dipterological Research 15(1): 57–62

[B11] Kahanpää J Winqvist K (2005) Check-list of Finnish flies: families Xylophagidae–Microphoridae. Sahlbergia 10(1): 10–27

[B12] Negrobov OP (1967) New Palaearctic species of the subfamily Medeterinae (Diptera, Dolichopodidae). Entomologicheskoe Obozrenie 46(4): 890–908

[B13] Negrobov OP Stackelberg AA (1972) 29. Dolichopodidae. In: Lindner E (Ed) Die Fliegen der palaearktischen Region 4(5). E. Schweizerbart‘sche Verlagsbuchhandlung, Stuttgart, 257–302

[B14] Pape T Blagoderov V Mostovski MB (2011) Order Diptera Linnaeus, 1758. In: Zhang Z-Q (Ed) Animal biodiversity: An outline of higher-level classification and survey of taxonomic richness.Zootaxa 3148: 222–229. http://www.mapress.com/zootaxa/2011/f/zt03148p229.pdf 10.11646/zootaxa.3703.1.1 26146682

[B15] Pollet M (2004) Fauna Europaea: Dolichopodidae. In: Pape T Beuk P (Eds) Fauna Europaea: Diptera. Fauna Europaea version 1.0. http://www.faunaeur.org

[B16] Ringdahl O (1933) Översikt av de i Sverige funna arterna av släktet *Atalanta* Meig. (*Clinocera* Meig.) inom fam. Empididae. Entomologisk Tidskrift 54(3–4): 277–282

[B17] Sinclair BJ Shamshev IV (2012) World revision of *Iteaphila macquarti* group (Diptera: Empididae). Zootaxa 3561: 1–61

[B18] Shamshev IV Sinclair BJ (2009) Revision of the *Iteaphila setosa* group (Diptera: Empididae). European Journal of Entomology 106: 441–450. doi: 10.14411/eje.2009.055

[B19] Sinclair BJ Cumming JM (2006) The morphology, higher-level phylogeny and classification of the Empidoidea (Diptera). Zootaxa 1180: 1–172. http://www.mapress.com/zootaxa/2006f/zt01180p140.pdf

[B20] Yang D Zhang K Yao G Zhang J (2007) World catalog of Empididae (Insecta: Diptera). China Agricultural University Press, Beijing, 599 pp

[B21] Yang D Zhu YJ Wang MQ Zhang L (2006) World catalog of Dolichopodidae (Insecta: Diptera). China Agricultural University Press, Beijing, 704 pp [+44 pl.]

[B22] Zetterstedt JW (1838) Insecta Lapponica. Dipterologis Scandinaviae. Sect. 3: Diptera. Leopold Voss, Lipsiae, 477–868

[B23] Zetterstedt JW (1843) Diptera Scandinaviae. Disposita et descripta. Tomus primus. Officina Lundbergiana, Lund, 894 pp

